# Functional properties of *in vitro* excitatory cortical neurons derived from human pluripotent stem cells

**DOI:** 10.1113/JP270660

**Published:** 2015-12-30

**Authors:** Matthew R. Livesey, Dario Magnani, Giles E. Hardingham, Siddharthan Chandran, David J. A. Wyllie

**Affiliations:** ^1^ Centre for Integrative PhysiologyUniversity of EdinburghEdinburghEH8 9XDUK; ^2^ Euan MacDonald Centre for MND ResearchUniversity of EdinburghEdinburghEH16 4SBUK; ^3^ Centre for Clinical Brain SciencesUniversity of EdinburghEdinburghEH16 4SBUK; ^4^ MRC Centre for Regenerative MedicineUniversity of EdinburghEdinburghEH16 4SBUK; ^5^ Centre for Brain Development and RepairInstitute for Stem Cell Biology and Regenerative MedicineBangalore560065India

## Abstract

The *in vitro* derivation of regionally defined human neuron types from patient‐derived stem cells is now established as a resource to investigate human development and disease. Characterization of such neurons initially focused on the expression of developmentally regulated transcription factors and neural markers, in conjunction with the development of protocols to direct and chart the fate of differentiated neurons. However, crucial to the understanding and exploitation of this technology is to determine the degree to which neurons recapitulate the key functional features exhibited by their native counterparts, essential for determining their usefulness in modelling human physiology and disease *in vitro*. Here, we review the emerging data concerning functional properties of human pluripotent stem cell‐derived excitatory cortical neurons, in the context of both maturation and regional specificity.

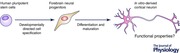

**Abbreviations**
AMPAR: AMPA receptor
GABA_A_R: GABA type‐A receptor
GlyR: strychnine‐sensitive glycine receptor
hPSC: human pluripotent stem cell
hPSC^C^ neuron: hPSC‐derived excitatory cortical neuron
LGIC: ligand‐gated ion channel
NMDAR: NMDA receptor

## Introduction

The human cerebral cortex controls higher cognitive abilities, including those that distinguish human beings from other mammalian species such as abstract thinking and complex language (Defelipe, 2011). This highly complex structure consists of several distinct areas that are responsible for processing and integrating different motor and sensory information and information stored in memory. Further functional and anatomical sophistication is reflected in the diversity and number of excitatory and inhibitory cell types, each characterized by distinct gene expression, morphology and functional properties (Molyneaux *et al*. 2007; Lui *et al*. 2011). The cortex is a target for many disorders of the brain at all stages of life. For example, perturbation of cortical development can lead to neurodevelopmental disorders, such as autism spectrum disorders, while the adult cortex is a major site for certain neurodegenerative conditions including Alzheimer's disease, frontotemporal dementia and Huntington's disease.

The majority of studies investigating cortical development and neuronal function have been based upon rodent models, but several aspects of the human cortex differ greatly from rodents including tangential expansion in the frontal cortex and gyrencephaly (Defelipe, 2011; Lui *et al*. 2011). Recently many laboratories have established *in vitro* protocols to derive excitatory pyramidal neurons, the principal neuronal type in the adult cortex, from human pluripotent stem cells (hPSCs) that provide powerful, readily accessible tools to model neuronal function in both healthy and disease contexts (Hansen *et al*. 2011; van den Ameele *et al*. 2014).

The ability of neurons derived from mouse and more recently from human stem cell lines to exhibit classical neuronal functional properties both *in vitro* and when integrated into host systems has been studied for many years (Benninger *et al*. 2003; Wernig *et al*. 2004). Increasing refinement and control in the derivation of neurons generated from embryonic or induced pluripotent stem cells (ESCs/iPSCs) now means it is possible to derive regionally specific neurons, including hPSC‐derived excitatory cortical neurons (hereafter termed ‘hPSC^C^ neurons’). This raises the question as to the extent such cells are able to recapitulate known details of native cortical development and ultimately whether they are appropriate models of ‘diseases in a dish’ (Sandoe & Eggan, 2013). In addition to forming synaptic connections to generate the intricate circuitry responsible for complex cortical processes, native excitatory cortical neurons undergo distinctive developmental changes in ion channel expression and ionic gradients that determine their function within cortical networks (Moody & Bosma, 2005). This review will principally focus on the emerging data examining the functional capability of hPSC^C^ neurons to exhibit known native‐like properties with regard to functional maturation and regional specification.

## Summary of cortical development

The cerebral cortex organizes in a complex 3‐dimensional structure comprising several anatomically distinct cortical areas. Cortical regionalization within the developing telencephalon is determined by graded expression of transcription factors expressed by cortical progenitor cells, including Gli3, Emx2, Pax6, Sp8 and Coup‐TF1 (O'Leary *et al*. 2007). This, in turn, is established by three major telencephalic signalling centres: the cortical hem that secretes Wnt and bone morphogenetic protein, the anterior neural ridge that secretes Fgf8, and the ventral telencephalon that secretes sonic hedgehog (Shh). Once the major cortical axes have been established, cortical progenitor cells generate neurons through the process of cortical lamination in a time‐dependent manner (Gaspard & Vanderhaeghen, 2011). During this process, later‐born neurons migrate over the early‐born neurons within the cortical plate forming a layered structure in an inside‐out fashion. Therefore, the deepest cortical layer VI forms first and the upper layer II last; the marginal zone or layer I, containing reelin^+^ Cajal–Retzius cells, escapes this inside‐out process (Frotscher, 1998). The adult cortex has a structure made up of six defined layers (I–VI) of diverse excitatory cortical neuron types that can be identified by layer‐specific markers, including CUX1 (layer II/III), ROR‐β (layer IV), CTIP2 (layer V), SOX5 (layers V and VI) and TBR1 (layer VI) (Molyneaux *et al*. 2007).

The correct time‐dependent patterning of the telencephalon and the formation of different neuronal subtypes within the different layers are ultimately essential for the appropriate functional wiring of cortical neurons with other cortical or subcortical targets (Germain *et al*. 2013). While neocortical excitatory pyramidal neurons form from distinct populations of cortical progenitor cells within the embryonic dorsal telencephalon, the majority of inhibitory interneurons originate in the ventral telencephalon and subsequently tangentially migrate into the developing cortex (Hansen *et al*. 2013).

## Specification of *in vitro* hPSC^C^ cultures

*In vitro*‐derived cortical progenitor cells giving rise to hPSC^C^ neurons can be formed from the spontaneous neuralization of hPSCs (Eiraku *et al*. 2008; Li *et al*. 2009; Mariani *et al*. 2012; Shi *et al*. 2012) and neuralization can be accelerated by using inhibitors of Wnt and bone morphogenetic protein/NODAL signalling pathways (dual‐SMAD inhibition; Chambers *et al*. 2009). Moreover, the presence of Shh or Shh agonists can give rise to ventral telencephalic progenitors from which cortical interneurons can differentiate (Germain *et al*. 2013; Maroof *et al*. 2013). Furthermore, hPSC‐derived cortical progenitors generally acquire a caudal identity by default as shown by their pattern of projections when transplanted in mouse brains (Espuny‐Camacho *et al*. 2013), yet they can be patterned to different cortical regions and respond to signalling cues when treated with morphogen agonists (Eiraku *et al*. 2008; Espuny‐Camacho *et al*. 2013; Kadoshima *et al*. 2013). Moreover, whilst the temporal generation of neurons belonging to different layers is largely maintained *in vitro* and the presence of neurons belonging to all six layers has been reported, the contribution of each layer considerably varies depending on the method used (see van den Ameele *et al*. 2014).

In these respects, *in vitro* neuronal connections and circuitry will therefore be somewhat limited by the protocol to recapitulate cortical development in its cellular specification and organization, particularly in monolayer cultures. However, aspects of cortical cytoarchitecture are remarkably maintained *in vitro*, in particular in 3‐dimentional cultures that allow the radial localization of later‐born neurons above earlier‐born ones (Mariani *et al*. 2012; Lancaster *et al*. 2013; Paşca *et al*. 2015).

Transcriptome analyses of the sequential phases of *in vitro* hPSC‐derived corticogenesis in monolayer or 3‐D culture demonstrate the ability of these protocols to reproduce gene expression profiles in hPSC^C^ neuron populations that are equivalent to early native human embryonic cortical development (Stein *et al*. 2014; Paşca *et al*. 2015). Importantly, the heterogeneity in the cellular specification of previously published protocols is also matched at the transcriptome level, where some protocols better recapitulate native development than others and, furthermore, have variable rates of hPSC^C^ neuron maturation (Stein *et al*. 2014). These data, however, do not directly examine the functional capacity of hPSC^C^ neurons. Many groups therefore apply patch‐clamp electrophysiology (and also live‐imaging) to assess directly the functional membrane properties of hPSC^C^ neurons.

## hPSC^C^ neurons are functionally reminiscent of immature cortical neurons

Neurons are defined by their excitable plasma membrane properties, which rely on the development of ionic gradients and the expression of ion channels. Reports demonstrating the generation of hPSC^C^ neurons have therefore focused upon basic intrinsic membrane properties and the ability of hPSC^C^ neurons to generate action potentials. Data indicate that intrinsic and action potential firing properties of hPSC^C^ neurons are broadly comparable to rodent cortical neurons at an embryonic/early postnatal stage of development (Johnson *et al*. 2007; Kim *et al*. 2011; Espuny‐Camacho *et al*. 2013; Bilican *et al*. 2014). Indeed, the majority of studies of hPSC‐derived neurons which have recorded electrophysiological properties consistently report input resistances that are 5‐ to 20‐fold higher than their adult, mature *in vivo* counterparts (Johnson *et al*. 2007; Kim *et al*. 2011; Espuny‐Camacho *et al*. 2013; Bilican *et al*. 2014). This is also true for hPSC‐derived neurons cultured for extended periods. Thus, hPSC‐derived neurons typically require only modest current injection to elicit action potential firing. hPSC^C^ neurons that show increases in excitability over time also demonstrate maturation in their expression of intrinsic membrane conductances that collectively define the nature of neuronal excitability (Johnson *et al*. 2007; Bilican *et al*. 2014). Additionally, interneuronal content is very low in some *in vitro* cultures (Shi *et al*. 2012; Bilican *et al*. 2014); however, many protocols do not report the extensiveness of interneuron differentiation in their cultures. It is important to note that interneuronal classes have highly variable mature firing properties (Markram *et al*. 2004) and as such this may be confused with firing patterns seen with immature excitatory neurons.

Studies have revealed that the resting membrane potential (RMP) of hPSC^C^ neurons hyperpolarizes with extended culture periods (Johnson *et al*. 2007; Bilican *et al*. 2014). Nevertheless RMPs can remain relatively depolarized and as such this compromises their ability to display spontaneous (TTX‐sensitive) action potential firing which is considered critical to the development and maturation of the cortex (Spitzer, 2006). Indeed, Weick *et al*. (2009) demonstrated that TTX only weakly blocked spontaneous Ca^2+^ transients in hPSC^C^ neuron cultures indicating that these transients were largely not mediated by spontaneous action potential activity. Careful pharmacological work in this study determined the source of Ca^2+^ to be mediated through L‐type voltage‐gated Ca^2+^ channels and transient receptor potential channels.

## Ligand‐gated ion channels

Ligand‐gated ion channels (LGICs) are integral to the process of fast neurotransmitter signalling and their activities contribute to the fine balance of excitation and inhibition within the CNS. LGICs are multimeric protein complexes that can comprise numerous subunit combinations which impose distinct biophysical and pharmacological properties. LGIC subunit composition is often regulated both developmentally and spatially. Thus, the assessment of LGIC composition in hPSC^C^ neurons is essential if we are to determine the extent to which these cells reflect native properties.

## Ionotropic glutamate receptors

Ionotropic glutamate receptors are the central mediators of fast excitatory neurotransmission in the cortex and are a family of three tetrameric receptor types: AMPA, NMDA and kainate receptors (Traynelis *et al*. 2010). NMDA receptors (NMDARs) are composed of two ubiquitously expressed GluN1 subunits and two potential GluN2A, GluN2B, GluN2C, GluN2D and/or GluN3A, GluN3B subunits (Wyllie *et al*. 2013). Considerable evidence shows that NMDARs in embryonic mammalian cortical neurons contain predominantly GluN1 and GluN2B subunits while maturation is associated with a functional up‐regulation of GluN2A subunits (Wyllie *et al*. 2013). For rodents this is a postnatal event; however, determining this in humans has proved challenging (Henson *et al*. 2008). hPSC^C^ neurons maintained in culture for 5 weeks express GluN1/GluN2B NMDARs as assessed by their sensitivity to the GluN2B‐selective antagonist ifenprodil and therefore an immature NMDAR profile (Livesey *et al*. 2013).

AMPA receptors (AMPARs) can be composed of GluA1, GluA2, GluA3 and GluA4 subunits of which the functional up‐regulation of the GluA2 subunit is associated with cortical neuronal maturation (Traynelis *et al*. 2010). Furthermore, GluA2 subunits predominantly undergo post‐transcriptional modification resulting in an arginine codon (GluA2(R)) replacing a glutamine codon (GluA2(Q)) in the M2 pore‐forming region of the channel. Therefore native cortical maturation is associated with a shift from GluA2(R)‐lacking to GluA2(R)‐containing AMPARs. Importantly, the presence of one or more GluA2(R) subunit in an AMPAR complex results in reduced single‐channel conductance, reduced sensitivity to channel‐blocking polyamines and, crucially, reduced Ca^2+^ permeability (Traynelis *et al*. 2010). Assessment of the functional AMPAR composition in hPSC^C^ neurons using non‐stationary fluctuation analysis to estimate mean AMPAR single‐channel conductance and their sensitivity to a GluA2(R)‐lacking AMPAR channel blocker indicates an activity‐independent and native‐like maturation from GluA2(R)‐lacking to GluA2(R)‐containing AMPARs within 5 weeks of *in vitro* differentiation (Livesey *et al*. 2014). GluA2 transcript expression also increases with time in culture (Chander & Weick, 2014; Stein *et al*. 2014; van de Leemput *et al*. 2014). Thus AMPAR expression in hPSC^C^ neurons appears to display properties that are observed in native mature neuronal populations (Isaac *et al*. 2007). Both NMDARs and AMPARs are expected to undergo maturational changes in composition in the early postnatal weeks of cortical development in rodents (Traynelis *et al*. 2010) and in this regard the ontogenetic development of AMPARs in hPSC^C^ neurons is much more rapid than expected. Interestingly, the GluA2 subunit has been shown to be rapidly edited and functionally up‐regulated 4 weeks after the *in vitro* differentiation of neurons from primary human cortical progenitors (Whitney *et al*. 2008) in contrast to the expected longer *in vivo* developmental time scales (Talos *et al*. 2006).

These data suggest the rapid maturation of the AMPAR complex is a potential product of the *in vitro* environment. Nonetheless, this feature provides an opportunity to examine numerous scenarios in which abnormal regulation of the GluA2 subunit is hypothesized or prevalent in adult human disease (Wright & Vissel, 2012).

## Ionotropic GABA and glycine receptors

GABA_A_ receptors (GABA_A_Rs) and strychnine‐sensitive glycine receptors (GlyRs) are pentameric LGICs that primarily mediate fast inhibitory neurotransmission in the mature cortex. GABA_A_Rs can be potentially composed of 19 known subunits (α1–6, β1–3, γ1–3, δ, ε, π, θ and ρ1–3) giving a vast number of theoretical possible GABA_A_R arrangements. Whilst in reality composition is tightly regulated, the breadth of possible GABA_A_R composition generates considerable functional and pharmacological diversity across brain regions and cellular locations (Olsen & Sieghart, 2009). Using a pharmacological and RNA‐seq‐based approach hPSC^C^ neurons differentiated for 5 weeks were shown to express GABA_A_Rs that had a predominant α2/3β3γ2 composition (James *et al*. 2014). This is the most common GABA_A_R combination present in the embryonic cortex (Olsen & Sieghart, 2009). Comparison of GABA_A_R subunit transcript levels in hPSC^C^ neurons with data from human primary tissue indicates that overall GABA_A_R subunit expression is similar to that seen in the cortex at 12–21 weeks postconception. Again this is in broad agreement with other transcriptome‐based studies (Stein *et al*. 2014; Paşca *et al*. 2015). Finally, pharmacological assessment of GABA_A_Rs in hPSC^C^ neurons is consistent with the absence of the α1‐subunit which is associated with more developmentally mature cortical neurons.

GlyRs are thought to play an important role in cortical development and transient functional GlyR expression is a feature of neocortical development in rodents (Flint *et al*. 1998; Avila *et al*. 2013). Indeed, hPSC^C^ neurons respond robustly to glycine application (James *et al*. 2014). Pharmacological and RNA‐seq analysis of GlyRs indicates that the GlyR composition is principally α2/β‐containing (James *et al*. 2014). Transcript levels indicate a level of maturity equal to that of GABA_A_Rs; however, it is thought that the early mammalian embryonic GlyR composition consists of homomeric α2 GlyRs and matures to α1/β‐containing GlyRs (Lynch, 2009). Interestingly, a transient GlyR population of α2/β‐containing GlyRs has been observed in developing rodent Cajal–Retzius cells (Okabe *et al*. 2004). hPSC^C^ neurons may prove useful in elucidating the role of GlyRs within the developing human cortex.

## Intracellular chloride

GABA_A_Rs and GlyRs are permeable to Cl^−^ ions and mediate inhibitory responses in adult cortical neurons. However, the application of either GABA or glycine to embryonic excitatory cortical neurons generates depolarizing excitatory responses due to elevated levels of intracellular Cl^−^ (Ben‐Ari *et al*. 2007). The developmental reduction in intracellular Cl^−^ concentration is a crucial feature of cortical development and its perturbation is implicated in numerous disease mechanisms (Blaesse *et al*. 2009).

Intracellular Cl^−^ activity in hPSC^C^ neurons (and neural precursor cells) measured using the perforated patch‐clamp technique falls from around 25 mm after 7 weeks in culture to <7 mm in hPSC^C^ neurons (Livesey *et al*. 2014). Correspondingly, the expression of K^+^–Cl^−^ cotransporter‐2 (KCC2, responsible for Cl^−^ efflux) increases while that of the Na^+^–K^+^–Cl^−^ cotransporter‐1 (NKCC1, responsible for Cl^−^ influx) falls in hPSC^C^ neurons, which is in agreement with native developmental mechanisms that regulate intracellular Cl^−^ activity (Ben‐Ari *et al*. 2007). In agreement with our data, Shcheglovitov *et al*. (2013) also report that application of GABA to hPSC^C^ neurons generates hyperpolarizing currents consistent with a reduction in intracellular chloride activity. The time of this switch has been reported with high variability in the human cortex (Blaesse *et al*. 2009), though it is interesting that a population of Pax‐6^+^ neural precursors give rise to subplate neurons that highly express KCC2 by 16 weeks postconception (Bayatti *et al*. 2008) and that Livesey *et al*. (2014) studied hPSC^C^ neurons from predominantly Pax‐6^+^ neural precursors. Livesey *et al*. (2014) also highlight that the use of neurotrophic media supplements influences the development of expression of Cl^−^ transporters and Cl^−^ activity. Although neurotrophic factors are added to promote synaptogenesis and/or increase responsiveness to neurotransmitters in pluripotent stem cell (PSC)‐derived neuronal cultures (Copi *et al*. 2005; Bardy *et al*. 2015), neurotrophic factors have an important role in the regulation of Cl^−^ transporter expression (Blaesse *et al*. 2009). Beyond pharmacological considerations regarding the use of chronic neurotrophic factor media supplements (Frank *et al*. 1996), the impact of neurotrophic factors on PSC‐derived neuronal physiology needs to be carefully considered.

## Synaptic and network properties

In addition to harbouring excitable membranes, it is a defining feature of neurons to receive and generate synaptic signals. These exist in two general forms; phasic and tonic, and are both essential to the normal function of the CNS. The intricate and specific synaptic connectivity displayed by native cortical neurons is key to cortical network development and function (Spitzer, 2006). It is therefore critical to the development of *in vitro* hPSC^C^‐derived neurons to recapitulate native synaptic properties. The co‐localization of pre‐ and postsynaptic membrane‐associated scaffold proteins such as synaptophysin and PSD‐95, respectively, provide an indication of architectural synapse formation, but not functionality. Functional synaptic activity is generally detectable in standard cultures of *in vitro* hPSC^C^‐derived neurons where phasic ionotropic glutamatergic receptor‐ and GABA_A_R‐mediated activity has been observed. The latter property is determined by the culture protocol employed and its potential to generate GABA‐ergic interneurons. However, it is largely accepted that many standard *in vitro* hPSC protocols do not generate cultures that exhibit robust synaptic activity (Bardy *et al*. 2015). Studies examining synaptic properties therefore employ techniques to promote synaptic formation in their cultures.

Moreover, many groups now co‐culture hPSC^C^ neurons with primary rodent astrocytes, which also promotes synaptic function (Johnson *et al*. 2007; Kim *et al*. 2011; Shcheglovitov *et al*. 2013; Wen *et al*. 2014; Pak *et al*. 2015). The analysis of the field‐evoked postsynaptic events in co‐cultured neurons demonstrates that glutamate activates both fast AMPAR‐mediated and slower GluN1/GluN2B‐like NMDAR‐mediated events in control neurons (Shcheglovitov *et al*. 2013). Interestingly in this study, cell lines lacking the postsynaptic density protein SHANK3, which is reduced in expression in a neurodevelopmental disorder (Phelan‐McDermid syndrome), exhibit evoked NMDAR‐mediated postsynaptic currents that have faster decay kinetics, which may be consistent with an NMDAR population expressing both GluN2A and GluN2B NMDAR subunits. Alternatively, presynaptic neurotransmitter‐release dysfunction has been modelled in hPSC^C^ neurons derived from schizophrenia (mutant *DISC1*; Wen *et al*. 2014) and autism (mutant *NRXN1*; Pak *et al*. 2015) patients. Recently, Bardy *et al*. (2015) reported that relatively sparse synaptic activity observed in PSC‐derived neurons is due, in part, to the media in which the neurons are maintained. Their study utilized a culture medium that promoted synapse formation and concomitantly increased functional AMPAR‐mediated synaptic activity in PSC‐derived neurons co‐cultured with mouse astrocytes. Thus, hPSC^C^ neurons can recapitulate synaptic activity and offer the exciting potential to study synaptic dysfunction to elucidate disease mechanisms. Table 1 describes reports of advanced culture techniques in order to study synaptic physiology.

**Table 1:** **A**dvanced culture techniques using hPSC^C^ neurons (or other hPSC‐derived neurons) that promote functional synaptic formation and/or maturation

			Physiological details measured
Approach	Study	Advanced protocol details	from hPSC^C^ neurons
Astrocyte co‐culture	Johnson *et al*. 2007	Co‐culture with primary E14^a^ mouse astrocytes.	Accelerated initial functional synapse formation, but long‐term unaffected. Spontaneous postsynaptic currents blocked by AMPAR and GABA_A_R antagonists
	Shcheglovitov *et al*. 2013	Co‐culture with primary rat cortical astrocytes.	Spontaneous and evoked postsynaptic currents that consist of AMPAR, GABA_A_R and NMDAR components. Hyperpolarizing GABA_A_R responses. High *R*_IN_.
	Wen *et al*. 2014	Co‐culture with primary rat astrocytes.	Spontaneous postsynaptic currents. Neurotransmitter release investigated using FM1‐43 imaging.
	Pak *et al*. 2015	Co‐culture with primary mouse astrocytes.	Evoked AMPAR‐mediated postsynaptic currents. High *R*_IN_.
Neuron co‐culture	Kim *et al*. 2011	Co‐culture with primary E18 rat cortical neurons (or with rat astrocytes).	Spontaneous postsynaptic currents. High *R*_IN_, as indicated by low rheobase needed to elicit action potential firing.
	Weick *et al*. 2011	Co‐culture with primary E16 rat cortical neurons.	Neurons adopt bursting activity of mouse neurons. Light activation of channelrhodopsin‐transduced neurons induces AMPAR‐sensitive bursting in mouse neurons.
Advanced media composition + astrocyte co‐culture	Bardy *et al*. 2015	Custom media formulation including media supplements. hPSC‐derived neurons co‐cultured with primary mouse astrocytes.	Neurons maintained in new formulation exhibit increased frequency of AMPAR‐mediated postsynaptic currents, but not GABA_A_R‐mediated postsynaptic currents. High *R*_IN_.
3‐Dimensional culture	Lancaster *et al*. 2013	‘Cerebral organoid’ development.	TTX‐sensitive spontaneous activity detected using Ca^2+^ imaging. Increase in Ca^2+^ detection upon application of glutamate.
	Paşca *et al*. 2015	‘Cortical spheroid’ development equivalent to 19–24 weeks fetal development.	Spontaneous firing activity and evoked excitatory postsynaptic currents blocked by glutamate receptor antagonists. High *R*_IN_, as indicated by low rheobase needed to elicit action potential firing.
Integration	Weick *et al*. 2011	Integration of hPSC^C^ neurons into mouse (aged 2 months) hippocampus.	Light activation of channelrhodopsin‐transduced neurons induces synaptic events in adjacent mouse neurons.
	Espuny‐Camucho *et al*. 2013	Integration of hPSC^C^ neurons into embryonic mouse cortex. Assessed 9 months post‐integration after key developmental period.	Spontaneous firing activity and evoked postsynaptic currents that can be blocked by glutamate receptor antagonists. Low *R*_IN_.

a E14, embryonic day 14. *R*_IN_, input resistance.

Similarly in *in vitro* cultures, reports of robust network activity have not yet been reported widely. A potential factor in the inability to observe synchronous multi‐neuronal firing in many cultures may be a consequence of the extent to which GABA‐ergic interneurons are present or absent in cultures. GABA‐ergic signalling is established before that of glutamatergic‐signalling in early development and is thought to initiate primitive neural network activity (Ben‐Ari *et al*. 2007). Indeed, inhibitory GABA‐ergic interneurons are essential for maintaining the balanced activity of cortical neural circuits. GABA‐ergic synaptic activity has been detected within hPSC^C^ cultures in the form of spontaneous postsynaptic currents (Johnson *et al*. 2007; Wu *et al*. 2007; Shcheglovitov *et al*. 2013). However, a recent report has observed synchronized excitatory neurotransmitter‐driven network activity that resembles that of early‐stage cortical development and which occurs in cultures that contain a low percentage of interneurons and is insensitive to pharmacological blockade of GABA_A_Rs (Kirwan *et al*. 2015). A key study in the near future will therefore be the culture of defined mixtures of hPSC^C^ neurons and defined populations of GABA‐ergic interneurons. Notably, it has been shown that hPSC^C^ neurons can integrate into the network firing properties generated by primary mouse cells (Weick *et al*. 2011) and, furthermore, light‐stimulated hPSC‐derived neurons transduced with channelrhodopsin can influence the network activity of hippocampal organotypic slices (Piña‐Crespo *et al*. 2012).

## Future challenges: making mature neurons

The capacity of hPSC^C^ neurons to become physiologically relevant is their successful incorporation into a native system. As an initial step along this road Espuny‐Camucho *et al*. (2013) successfully demonstrated the differentiation and functional integration of hPSC^C^ neurons into rodent cortex. Several months after transplantation into the developing cortex hPSC^C^ neurons exhibited intrinsic membrane properties consistent with adult mature neurons, in contrast to the more immature properties of *in vitro* differentiated hPSC^C^ neurons. hPSC^C^ neurons incorporated into the rodent cortex also received synaptic inputs. Integration of hPSC^C^ neurons expressing channelrhodopsin to the mouse hippocampal CA1 region has demonstrated the ability to generate light‐evoked postsynaptic currents in adjacent (untransfected) neurons (Weick *et al*. 2011). These data indicate the potential of such neurons to contribute to cortical network activity. Furthermore, it has been reported that hPSC^C^ neurons integrated into a rat model of stroke promote functional recovery (Tornero *et al*. 2013). While there is clearly still much to learn with regard to *in vivo* functional integration, studies such as these give strong support to the notion that hPSC^C^ neurons have the capacity to possess functionally mature phenotypes. Similarly, hPSC^C^‐derived ventral telencephalic interneurons develop mature intrinsic properties and receive synaptic input when integrated into the embryonic rodent cortex (Nicholas *et al*. 2013). Furthermore, this study indicates that interneuron maturation can be achieved with co‐culture in the presence of rodent glia and requires extended culture periods (>6 months). An equivalent study has not been performed for hPSC^C^ excitatory neurons. These data indicate that *in vitro* hPSC‐derived neuron cultures lack important factors that are present *in vivo* and that are required for maturation.

Aside from their electrophysiological phenotype, hPSC^C^ neurons need to display morphological characteristics typical of native cortical neurons. Dendritic spines form the principal location at which excitatory synaptic transmission and synaptic plasticity take place and, moreover, numerous neurological diseases are accompanied by spine number or size alterations (Bourne & Harris, 2008; Penzes *et al*. 2011). In this respect it is of significance that *in vivo* integrated hPSC^C^ neurons do appear to develop spine‐like structures (Espuny‐Camucho *et al*. 2013), but dendritic spine structures with co‐localized expression of PSD‐95 are infrequent in *in vitro* hPSC‐derived neurons (Marchetto *et al*. 2010).

In conclusion, a major challenge is to generate neuronal populations that exhibit maturation profiles that more closely reflect those seen *in vivo*. Co‐culture with astrocytes and mixed neuronal populations, together with the maintenance of cells in media that promote increased synaptic activity, indicates that such strategies are required to assess synaptic transmission. When achieved, this will enhance and make more relevant our ability to study human physiology and pathophysiology using *in vitro* hPSC^C^ neurons.

## Additional information

### Competing interests

None declared.

### Funding

Aspects of work described in this review article were funded by The Wellcome Trust (Grant 092742/Z/10/Z to D.J.A.W., G.E.H. and S.C.), the Motor Neurone Disease Association (MNDA) (S.C.), the Euan MacDonald Centre (S.C.) the Medical Research Council (Grant to MR/J004367/1 to S.C.), NC3Rs (Grant CRACK IT to S.C.) and a Seedcorn grant from the Patrick Wild Centre/RS Macdonald Trust (D.J.A.W.). M.R.L. holds a Royal Society of Edinburgh Personal Research Fellowship funded by the Caledonian Research Fund.
